# Metastatic Seminoma with Positive Staining of Cytokeratin and MOC31: A Diagnostic Pitfall

**DOI:** 10.1155/2021/9992978

**Published:** 2021-06-29

**Authors:** Jiaming Fan, Ren Yuan, David Stefanelli, Gang Wang

**Affiliations:** ^1^Tianjin Medical University, Tianjin, China; ^2^Department of Radiology, British Columbia Cancer Vancouver Centre, Vancouver, BC, Canada; ^3^Department of Pathology, Royal Inland Hospital, Kamloops, BC, Canada; ^4^Department of Pathology, British Columbia Cancer Vancouver Centre, Vancouver, BC, Canada

## Abstract

Retroperitoneal metastasis of seminoma often occurs in the higher stage through lymph nodes. Generally, seminoma expresses specific germ cell markers while being negative for carcinoma markers. We present a unique case of cytokeratin positive seminoma initially presented as retroperitoneal metastasis. The diagnosis was made based on the histological features and immunohistochemical stains. Testicular ultrasound confirmed the primary tumor in the patient's left testicle. Pathologists should always be aware of germ cell tumors when encountering a metastasis of an unknown primary.

## 1. Introduction

Testicular germ cell tumors are composed of seminomas and nonseminomas. Seminoma accounts for more than half of the diagnosis of germ cell tumors [1]. Most seminomas are localized to the testis, but some tend to present with lymph node metastases, mainly to the retroperitoneum [2]. Although in some cases, the morphology of retroperitoneal metastasis shows a little difference with the tumor in the primary site, and the metastatic and primary tumors share similar germ cell features such as positivity of tumor markers OCT3/4, CD117, and PLAP [3]. There are few reports of other epithelial or vascular markers being positive in seminoma [4]. Epithelial markers like pan-cytokeratin, MOC31 often implicate that tumors have epithelial origin or epithelial differentiation tendency, so they have been applied to diagnose carcinomas. Here, we report a unique case of metastatic seminoma in the retroperitoneum with positive staining of cytokeratin and MOC31.

## 2. Case Presentation

**Figure 1:**
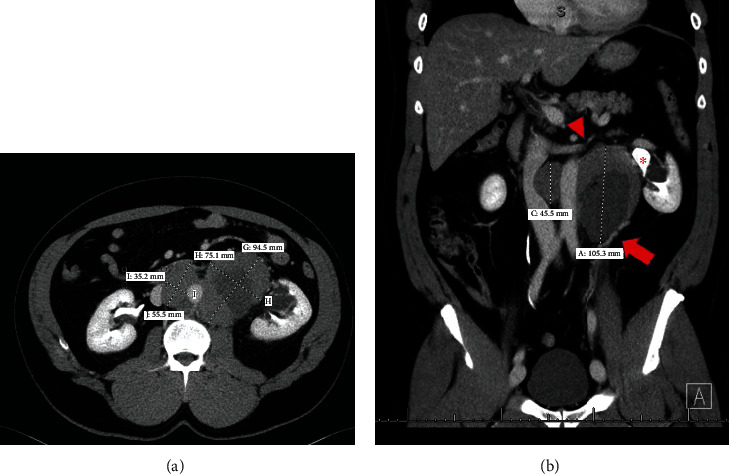
(a) Conglomerating bilateral paraaortic nodal mass circumferentially encases without narrowing the aorta. (b) The large left-side mass measures 9.5 × 7.5 × 10.5 cm containing a large internal necrosis. It is inseparable from the left gonadal vessels (arrow), superiorly extends to the renal vein level (arrowhead), and laterally it displaced the kidney and renal pelvis (^∗^) causing mild hydronephrosis. The 3.5 × 5.5 × 4.5 cm right-side mass indents the inferior vena cava.

A 52-year-old man complained about severe lower abdominal pain for days but without any digestive symptoms such as nausea, vomiting, diarrhea, or constipation, as well as any thoracic discomforts. The abdominal CT showed a 12.1 × 10.8 × 8.6 cm mass encasing the aorta circumferentially in the retroperitoneum (Figure 1). Containing considerable internal necrosis, the 9.5 × 7.5 × 10.5 cm left-side mass displaced the left kidney and renal pelvis, which caused mild left hydronephrosis. In comparison, the 3.5 × 5.5 × 4.5 cm right-side one squeezed the inferior vena cava slightly. Then, a core biopsy was taken from the sizeable left retroperitoneal mass.

**Figure 2:**
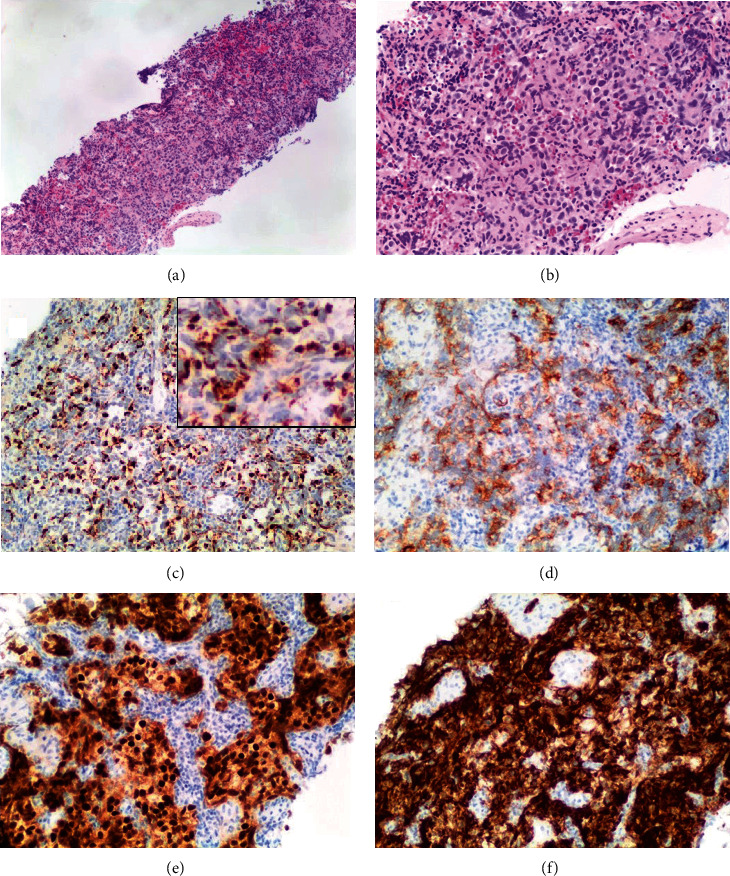
(a) Low power view infiltrative tumor cells mixed with lymphocytes. (b) High power view showing the infiltrating tumor cells with a sheet-like growth pattern with medium to large size, pale to clear cytoplasm, and polyclonal nuclei. (c) The tumor cells were positive for pan-cytokeratin. (d) The tumor cells are positive for MOC31. (e) The tumor cells were diffusely and strongly positive for Oct-3/4. (f) The tumor cells were diffusely and strongly positive for CD117.

Microscopic examination revealed that the lesion was comprised of infiltrative malignant cells mixed with lymphocytes (Figure 2(a)). The infiltrative tumor cells displayed a sheet-like growth pattern with medium to large size, pale to clear cytoplasm, and polyclonal nuclei. Some of them were undergoing mitosis (Figure 2(b)). The immunostains showed that the tumor cells were negative for lymphoid and organ-specific markers, including CD3, CD20, CD45, CD30, S100, GATA3, calretinin, PAX8, TTF-1, CK7, CK20, CDX2, synaptophysin, chromogranin, and NKX3.1. The malignant tumor showed positive for pan-cytokeratin (Figure 2(c)) and MOC31 (Figure 2(d)). In comparison, the cells were diffusely and strongly positive for OCT-3/4 (Figure 2(e)) and CD117 (Figure 2(f)) which are the markers of germ cells. Ki-67 stained about 70% to 80% of the tumor cell nuclei. The overall immunostaining findings were consistent with metastatic seminoma, classic type.

**Figure 3:**
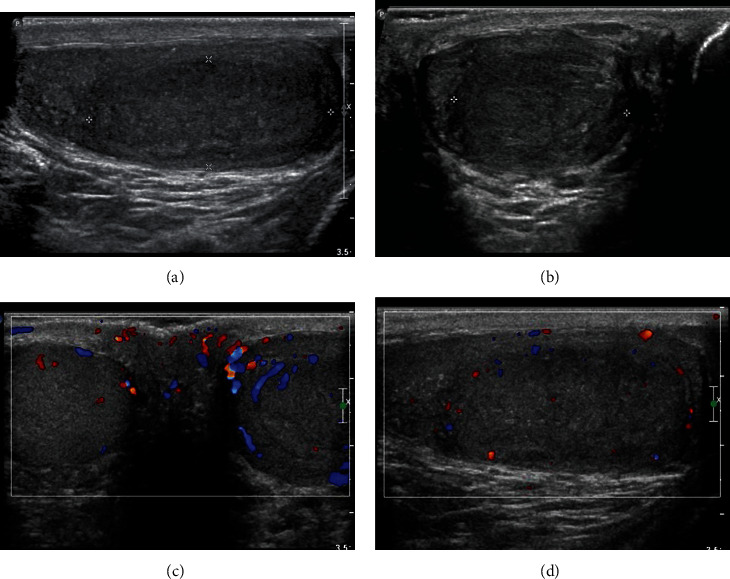
(a, b) US demonstrate a 2.4 × 1.6 × 3.7 cm left intratesticular heterogenous hypoechoic mass ((a) sagittal view; (b) transverse view). (c) Color Doppler transverse view demonstrates asymmetrical enlargement of the left testicle (right side) containing a heterogeneous mass, compare to the right testicle (right side). (d) Mild internal vascularity of the mass in the left testicle.

Since the retroperitoneal biopsy suggested a metastatic seminoma, a further image study was done. Testicular ultrasound revealed a 2.4 × 3.7 × 1.6 cm heterogeneous hypoechoic mass in the left testicle (Figures 3(a) and 3(b)). The Color Doppler showed asymmetrical enlargement of the left testicle containing a hypervascular tissue mass compared to the right testicle (Figures 3(c) and 3(d)). This testicular mass was considered the primary lesion of the classic seminoma. The patient's serum tumor markers (LDH, hCG, AFP) were all in the normal range.

After completing curative intent chemotherapy with four cycles of cisplatin etoposide, the patient underwent left radical orchiectomy. The pathology sections demonstrated a nodular scar confined to the testis but with the involvement of rete testis. The background testicular parenchyma showed significant atrophy of the seminiferous tubules. There was no residual germ cell tumor or germ cell neoplasia in situ identified.

## 3. Discussion

Seminoma tends to have retroperitoneal or mediastinal metastasis through lymph nodes [5]. The gross pathologic analysis reports that the classic seminoma is fleshy, solid, lobular, tan to pale yellow or gray-white [3, 6]. At the histological examination, seminoma shows a nest-like or sheet-like growth mode with large polygonal nuclei, pale to clear to eosinophilic cytoplasm, distinct cell membrane, and intervening thin fibrous septa. The septa may be composed of lymphocytes [3]. However, the histological morphology is a little unusual in some metastasis cases [7], so the diagnosis of seminoma should include the morphological assessment and the immunohistochemical evaluation to separate other tumors [5]. Seminoma is usually positive for the staining of germ cell markers, including OCT3/4, CD117, PLAP, SOX17, SALL4, and D2-40 [3, 6, 8]. Some testicular tumor markers (LDH, *β*-HCG, *α*-FP) can also be detected in classic seminomas [9], even in some cases of retroperitoneal metastasis [6]. Noticeably, seminoma typically has a negative immunoreactivity to other organs/lineage-specific markers [10]. However, here, we report a case of classic seminoma showing positive for cytokeratin and MOC31.

Commonly, cytokeratin can be widely detected in the normal epithelium by AE1/AE3, the pan-cytokeratin monoclonal antibody [7, 11]. Cytokeratin is a significant component of intermediate filaments in epithelial cells, and they almost account for 80% of the total protein content of stratified epithelia [12, 13]. Hence, cytokeratin expression's positive results in a tumor are always considered a symbol of epithelial origin. Similarly, MOC-31 is another common epithelial marker frequently applied in clinical practice [14, 15]. As a kind of cluster that can recognize a transmembrane glycoprotein of cells [16], MOC-31 could react with normal epithelia and adenocarcinomas but not with mesotheliomas [17, 18], which is helpful in differential diagnosis. Hence, in the present case, these two markers' positivity may lead to a misdiagnosis of metastatic carcinoma.

Once the germ cell origin of this metastasis was established, we should exclude the nonseminomatous component, especially embryonal carcinoma. Indeed, we noticed that some tumor cells showed increased nuclear pleomorphism and cell crowding, making it hard to distinguish from embryonal carcinoma in the morphology [19]. On the other hand, both seminoma and embryonal carcinoma show similar patterns of immunohistochemical OCT3/4 expression [20]. While embryonal carcinoma is usually strongly positive for cytokeratin, once classic seminoma shows positivity for cytokeratin markers like in the current case, it would be difficult to distinguish between them. In this dilemma, other germ cell markers would be useful. CD117 should be expressed in classic seminoma while negative in embryonal carcinomas, while CD30 should be positive in embryonal carcinomas but negative in seminomas [4, 7, 19, 21, 22].

Interestingly, the staining results of two markers in the case we present may suggest epithelial differentiation in seminoma. The incidences of classic seminoma being positive for any epithelial marker were between 39% and 48%, usually weak and focal [22]. Kommoss et al. reported HEA125 (a different clone of EpCAM from MOC31) immunoreactivity in 3/12 testicular seminomas, some of which are diffuse and strong [23]. Cheville et al. revealed that seminoma could express cytokeratins of stratified epithelia [21]. Tickoo et al. demonstrated that expressing cytokeratin might have been “seminomas with atypia,” a subset of seminomas presenting at a higher clinical stage [4]. However, another study showed no differences in patient age, stage, tumor size, or outcome between CK-positive and CK-negative seminomas [21]. Currently, there is no convincing evidence implying that seminoma with epithelial differentiation has more aggressive behavior. The staining patterns of cytokeratin in seminoma vary among the reported cases, ranging from a dot-like pattern, cytoplasmic, to membranous staining, depending on different labs and antibodies. The current case showed globular and dot-like staining for pan-CK and moderate cytoplasmic staining for MOC31. More cases need to be studied to investigate whether there is any particular staining pattern(s) of cytokeratin associated with classic seminoma.

In summary, pathologists should be aware that some classic seminoma may express epithelial markers, including pan-cytokeratin and MOC31. When dealing with metastasis with unknown primary, epithelial markers' positivity should not exclude the possibility of germ cell tumors, including classic seminoma.

## Data Availability

The data used to support the findings of this study are included within the article.

## References

[1] Durer C., Comba I. Y., Durer S., Torres Luna N., Jignesh P., Carilli A. (2020). Seminoma metastasized to the prostate: a case report and literature review. *Urology Case Reports*.

[2] Dieker C. A., Davis B. R., de Las Casas L. E. (2013). Retroperitoneal metastatic germ cell tumor presenting as a psoas abscess: a diagnostic pitfall. *The American Journal of the Medical Sciences*.

[3] Marko J., Wolfman D. J., Aubin A. L., Sesterhenn I. A. (2017). Testicular seminoma and its mimics: from the radiologic pathology archives. *Radiographics*.

[4] Tickoo S. K., Hutchinson B., Bacik J. (2002). Testicular seminoma: a clinicopathologic and immunohistochemical study of 105 cases with special reference to seminomas with atypical features. *International Journal of Surgical Pathology*.

[5] Schmoll H. J., Jordan K., Huddart R. (2009). Testicular seminoma: ESMO clinical recommendations for diagnosis, treatment and follow-up. *Annals of Oncology*.

[6] Gingu C. V., Mihai M., Baston C. (2016). Primary retroperitoneal seminoma - embryology, histopathology and treatment particularities. *Romanian Journal of Morphology and Embryology*.

[7] Sung M. T., MacLennan G. T., Cheng L. (2006). Retroperitoneal seminoma in limited biopsies: morphologic criteria and immunohistochemical findings in 30 cases. *The American Journal of Surgical Pathology*.

[8] Looijenga L. H. J., Van der Kwast T. H., Grignon D. (2020). Report from the International Society of Urological Pathology (ISUP) consultation conference on molecular pathology of urogenital cancers: IV: current and future utilization of molecular-genetic tests for testicular germ cell tumors. *The American Journal of Surgical Pathology*.

[9] Yuan R., Zhou C., Meneghetti V., Lavoie J. M., Kollmannsberger C., Wang G. (2020). Seminoma presenting as a solitary metastasis in gastric mucosa with regressed testicular mass. *Urology Case Reports*.

[10] Delahunt B., Eble J. N., King D., Bethwaite P. B., Nacey J. N., Thornton A. (2000). Immunohistochemical evidence for mesothelial origin of paratesticular adenomatoid tumour. *Histopathology*.

[11] Orchard G. E., Wojcik K., Shams F. (2015). Pan-cytokeratin markers for rapid frozen section immunocytochemistry from head and facial Mohs cases of basal cell carcinoma: a comparison and evaluation to determine the marker of choice. *British Journal of Biomedical Science*.

[12] Bragulla H. H., Homberger D. G. (2009). Structure and functions of keratin proteins in simple, stratified, keratinized and cornified epithelia. *Journal of Anatomy*.

[13] Pekny M., Lane E. B. (2007). Intermediate filaments and stress. *Experimental Cell Research*.

[14] Gill P., Naugler C., Abi Daoud M. S. (2019). Utility of Ber-EP4 and MOC-31 in basaloid skin tumor detection. *Applied Immunohistochemistry & Molecular Morphology*.

[15] Pan C. C., Chen P. C., Ho D. M. (2004). The diagnostic utility of MOC31, BerEP4, RCC marker and CD10 in the classification of renal cell carcinoma and renal oncocytoma: an immunohistochemical analysis of 328 cases. *Histopathology*.

[16] Porcell L A. I., de Young B. R., Proca D. M., Frankel W. L. (2000). Immunohistochemical analysis of hepatocellular and adenocarcinoma in the liver: MOC31 compares favorably with other putative markers. *Modern Pathology*.

[17] Proca D. M., Niemann T. H., Porcell A. I., DeYoung B. R. (2000). MOC31 immunoreactivity in primary and metastatic carcinoma of the liver: report of findings and review of other utilized markers. *Applied Immunohistochemistry*.

[18] Ruitenbeek T., Gouw A. S., Poppema S. (1994). Immunocytology of body cavity fluids. MOC-31, a monoclonal antibody discriminating between mesothelial and epithelial cells. *Archives of Pathology & Laboratory Medicine*.

[19] Lau S. K., Weiss L. M., Chu P. G. (2007). D2-40 immunohistochemistry in the differential diagnosis of seminoma and embryonal carcinoma: a comparative immunohistochemical study with KIT (CD117) and CD30. *Modern Pathology: an official journal of the United States and Canadian Academy of Pathology, Inc*.

[20] Jones T. D., Ulbright T. M., Eble J. N., Baldridge L. A., Cheng L. (2004). OCT4 staining in testicular tumors: a sensitive and specific marker for seminoma and embryonal carcinoma. *The American Journal of Surgical Pathology*.

[21] Cheville J. C., Rao S., Iczkowski K. A., Lohse C. M., Pankratz V. S. (2000). Cytokeratin expression in seminoma of the human testis. *American Journal of Clinical Pathology*.

[22] Sung M. T., Maclennan G. T., Lopez-Beltran A., Zhang S., Montironi R., Cheng L. (2008). Primary mediastinal seminoma: a comprehensive assessment integrated with histology, immunohistochemistry, and fluorescence in situ hybridization for chromosome 12p abnormalities in 23 cases. *The American Journal of Surgical Pathology*.

[23] Kommoss F., Oliva E., Bittinger F. (2000). Inhibin-*α*, CD99, HEA125, PLAP, and chromogranin immunoreactivity in testicular neoplasms and the androgen insensitivity syndrome. *Pathology*.

